# Transmission of Onchocerciasis in Wadelai Focus of Northwestern Uganda Has Been Interrupted and the Disease Eliminated

**DOI:** 10.1155/2012/748540

**Published:** 2012-08-26

**Authors:** Moses N. Katabarwa, Frank Walsh, Peace Habomugisha, Thomson L. Lakwo, Stella Agunyo, David W. Oguttu, Thomas R. Unnasch, Dickson Unoba, Edson Byamukama, Ephraim Tukesiga, Richard Ndyomugyenyi, Frank O. Richards

**Affiliations:** ^1^Emory University and The Carter Center, One Copenhill, 453 Freedom Parkway, Atlanta, GA 30307, USA; ^2^80 Arundel Road, Lythan St. Anne's, Lancashire FY8 1BN, UK; ^3^The Carter Center, Uganda, P.O. Box 12027, Kampala, Uganda; ^4^Vector Control Division, Ministry of Health, 15 Bombo Road, P.O. Box 1661, Kampala, Uganda; ^5^Global Health Infectious Disease Research, College of Public Health, University of South Florida 3720 Spectrum Boulevard, Suite 304, Tampa, FL 33612, USA; ^6^Nebbi District Health Services, P.O. Box 1, Nebbi, Uganda; ^7^Kabarole District Health Services, P.O. Box 38, Kabarole, Uganda

## Abstract

Wadelai, an isolated focus for onchocerciasis in northwest Uganda, was selected for piloting an onchocerciasis elimination strategy that was ultimately the precursor for countrywide onchocerciasis elimination policy. The Wadelai focus strategy was to increase ivermectin treatments from annual to semiannual frequency and expand geographic area in order to include communities with nodule rate of less than 20%. These communities had not been covered by the previous policy that sought to control onchocerciasis only as a public health problem. From 2006 to 2010, Wadelai program successfully attained ultimate treatment goal (UTG), treatment coverage of ≥90%, despite expanding from 19 to 34 communities and from 5,600 annual treatments to over 29,000 semiannual treatments. Evaluations in 2009 showed no microfilaria in skin snips of over 500 persons examined, and only 1 of 3011 children was IgG4 antibody positive to the OV16 recombinant antigen. No *Simulium* vectors were found, and their disappearance could have sped up interruption of transmission. Although twice-per-year treatment had an unclear role in interruption of transmission, the experience demonstrated that twice-per-year treatment is feasible in the Ugandan setting. The monitoring data support the conclusion that onchocerciasis has been eliminated from the Wadelai focus of Uganda.

## 1. Introduction

The Wadelai onchocerciasis focus is one of the smallest in Uganda, comprising only about 15,000 people living close to the lower River Ora in the Nebbi district. It is not clear when this focus first came to the attention of the health authorities, but in 1951 onchocerciasis was recognised in the upper reaches of the River Aroga (a major tributary of the River Ora). The vector was assumed to be *Simulium neavei* [1] based on the forested environment. Much more was learnt of the distribution of onchocerciasis and its vectors in the following two decades. The breeding of a non-man-biting form of *S. damnosum s.l*, was reported in 1966 along River Ali, located opposite Rhino Camp roughly 15 kilometres downstream of the River Ora outfall [2, 3]. Barnley in his lecture notes delivered at Makerere University in 1968 entitled “The Distribution of Onchocerciasis and its Vectors in Uganda”, confirmed that *S. neavei* was the vector in the upper reaches of River Ora system, but made no mention of the situation in its lower reaches where the Wadelai focus is located [4]. Later in the Uganda Atlas of disease distribution, Barnley gave a distribution map of onchocerciasis and its vectors showing the presence of a small onchocerciasis focus in Wadelai, and in the vicinity of the River Ora outfall in the Albert Nile transmitted by *S. damnosum s.l *[5]. As such, it became a target for piloting the elimination approach in late 2005.

## 2. Methods

### 2.1. Baseline Parasitological (Nodule and Skin Snips) Assessment in 1993 

#### 2.1.1. Nodule Assessment

 Rapid epidemiological mapping of onchocerciasis (REMO) and rapid epidemiological assessment (REA) was conducted by nodule palpation with the assistance of River Blindness Foundation (RBF) and WHO/TDR throughout Nebbi district of north-western Uganda including the Wadelai area [6–8]. Based on REMO protocol for community selection, only one community (Olimbuni/Aroga) was selected for mapping and as a sentinel site in 1993. REMO is where “high risk” communities are first identified at every 30 km along the river, and additional primary communities located 10 km away from “high risk” ones are selected. If warranted, then secondary communities 10 km away from primary communities and tertiary communities 10 km from secondary communities are selected until onchocercal nodule-free communities are reached. Assessment of nodule rates was done among 30 adults of at least 20 years of age who had lived in the community for 20 years or more [7]. The results were expressed as a proportion of the number of positive/negative persons in the sample. 

#### 2.1.2. Skin Snips Microfilariae (mf) Assessment

Skin snips were also obtained from 50 adults in the same community (Olimbuni/Aroga) before mass treatment. The tip of a sterile lancet needle mounted in a holder was used to elevate 3-4 mm of skin over the right posterior superior iliac crest after cleansing the skin with alcohol. A sterile surgical razor blade was then used to remove a skin snip at the base of the elevation. The skin, dangling from the tip of the needle, was transferred to a well of 96 microtiter plate containing sterile normal saline solution. The blade and needle were then used to obtain the second specimen on the left side in the same manner, after which the needle and blade were discarded in an appropriately safe “sharps” container [6, 9]. The use of a disposable razor and needle is a government policy in order to avoid transmission of communicable diseases such as HIV/AIDS and hepatitis, as well as providing the program with standard tools that are affordable and readily available in the country. The skin snips were kept at room temperature in the microtiter plate in normal saline solution for 12–24 h to allow any mf present to emerge from the skin. Each skin snip was then removed from the well with a needle, and the saline solution was examined unstained under a microscope (40x) for mf of *O. volvulus*. The results were expressed as a proportion of the number of positive/negative persons in the sample.

**Figure 1:**
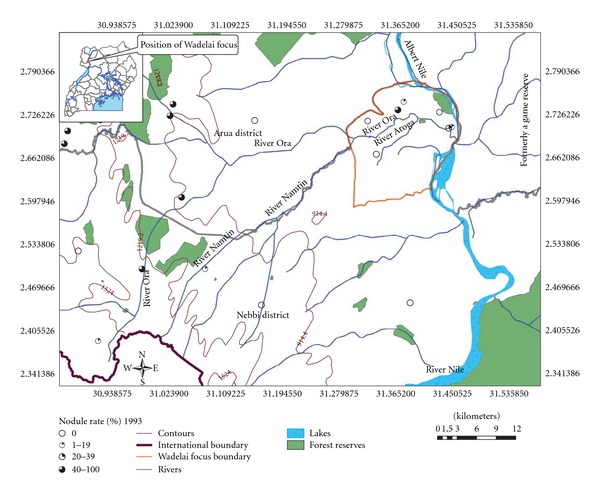
Wadelai focus 1993 rapid epidemiological assessment (REA) map of onchocerciaisis by nodule prevalence.

Parasitological (nodule palpation and skin snips) assessments were carried out during 1993 in Olimbuni/Aroga community, prior to annual mass treatment with ivermectin. Wadelai focus was demonstrated to be isolated from other onchocerciasis endemic communities in the area (see Figure 1 title below).

### 2.2. Mass Treatment

**Figure 2:**
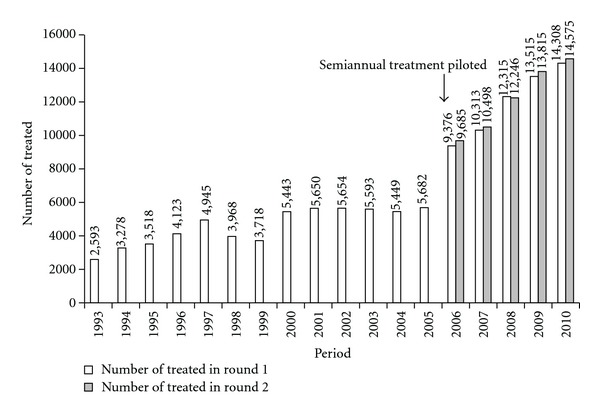
History of mass treatment with ivermectin from 1993 to 2010 in Wadelai onchocerciasis focus, Uganda.

Annual mass treatment with ivermectin commenced in 1993 when 2,593 persons were treated. In 1993 community-based treatment was introduced with the support of the River Blindness Foundation and in 1999 communities were empowered to make their own decisions under community-directed treatment with ivermectin (CDTI) [10]. Under CDTI, treatments grew to 5682 in 19 communities by 2005. When elimination effort was piloted in 2006, semiannual (i.e. every six month) distribution was launched, and, in 2007, geographic coverage was expanded to include all 34 communities in Wadelai area encompassing communities with nodule rate less than 20% representing, “full geographic coverage” (Figure 2) [11]. Such communities were previously not considered for mass treatment with ivermectin under the policy for controlling onchocerciasis as a public health problem [12]. Therefore, launching of elimination policy resulted in a considerable expansion of treatments to over 29,000 by 2010. In spite of the change from a single annual dose to semi-annual dose of ivermectin, ultimate treatment goal (UTG) was attained every year. UTG is the sum of all eligible persons for treatment (minus children <5 years) among the total number of people at risk living in all at-risk communities in the onchocerciasis endemic area that the program ultimately has to treat [13]. The UTG for Wadelai twice-per-year treatment was determined to be 30,000. In twice yearly treatments, the UTG doubles, and therefore is the number of ivermectin treatments, and not people.

### 2.3. 2008–2010 Follow-Up Assessments of Wadelai

#### 2.3.1. Parasitological Assessments

Baseline standard skin snip (microscopy) and nodule palpation from Olimbuni/Aroga community were compared with follow up data in 2009. In the follow up survey, 513 adults and children from six communities including Olimbuni/Aroga were assessed [6, 9]. Nodules were excised from six willing resident volunteers, sectioned, and stained by H&E to allow for evaluation of the presence and fertility of *O. volvulus *worms [14]. All the six persons were born and lived in Wadelai area. The national guidelines stipulate that elimination of morbidity is considered attained when the microfilaria prevalence in skin snips is less than 5% in all sampled communities, and less than 1% in 90% of sampled communities [15]. 

#### 2.3.2. Serological Assessments

The prevalence of IgG4 antibodies to OV-16, a recombinant antigen of *O. volvulus*, previously applied in school-age children [16], was used in all children 3 to ≤14 years of age in the entire Wadelai focus. A sample size of at least 3,000 schoolchildren is needed to calculate a prevalence rate with a one-sided 95% CI <0.1% if zero positives are found [16]. Meetings were held with community members, teachers, and community leaders, in order to explain the purpose of taking blood spots from the children's finger tips. A sample size of 3,011 resident schoolchildren was included in the study, representing most of the school-age children born and raised in the Wadelai focus. ﻿The national guidelines based on WHO criteria consider onchocerciasis elimination has occurred when the prevalence of infection (defined as antibodies to OV-16) is <0.1%. The antibody (IgG4) is a marker of early exposure and specific for *Onchocerca volvulus,* and therefore can be used to detect the presence of *O. volvulus* in the early prepatent period of infection [17]. Its sensitivity was 76.5%–81.1% and specificity 100% [18]. Sterile procedures were used to collect blood samples through finger pricking, and four six drops of blood from each participants were absorbed onto Whatman No. 2 filter paper (Sigma). The filter paper blood samples were dried, separated by sheets of paper, systematically bundled, and stored in plastic bags in a cooler until they were returned to the laboratory and stored at 4°C before being processed for analysis. 

#### 2.3.3. Laboratory Analysis

Two 6 mm punches of saturated filter paper per person were placed in a phosphate-buffered saline (PBS)-Tween 0.05% and bovine serum albumin (BSA) 5% buffer and eluted overnight at 4°C. Each elution was run in duplicate in a standard enzyme-linked immunosorbent assay (ELISA) to detect IgG4 antibodies against the OV-16 recombinant antigen [16]. A standard curve on each plate to identify positive samples and permit comparisons between plates and over days was applied. The cut-off was chosen as 40 arbitrary units by identifying the value that optimized both sensitivity and specificity. All positive results were individually repeated from the stored blood spot before being reported as positive. Skin snips were collected from the children whose blood spots were positive and subjected to O-150 polymerase chain reaction (PCR) analysis in order to confirm presence of patent *O. volvulus* infection [19]. O-150 polymerase chain reaction (PCR) analysis is applied as a confirmatory test since it is 100% sensitive and 100% specific as it detects *O. volvulus* DNA, therefore, the infection, while OV16 antigen detects exposure [20, 21].

#### 2.3.4. Entomological Assessments

A rapid entomological survey was conducted in 2008 along the main River Ora, where five sites were surveyed. In February, 2010, a brief but intense search for *S. neavei* and *S. damnosum s.l* was made over two days along the main River Ora. On the third day of this effort, surveys upstream of River Ora within the focus at 3 sites were carried out. Subsequent landing fly catches made at 2 sites for 8 man days were also conducted. Throughout the focus, interviews with residents in order to determine if *Simulium* fly biting was occurring were also conducted. Upstream of the focus, only 11 crabs were caught at 2 sites in 12 hours trapping. None were infested with *S. neavei* larval stages.

#### 2.3.5. Ethical Review

Parasitological, serological, and entomological evaluations were approved from the Ministry of Health in Uganda and Emory Institutional Review Board classified them as periodical program performance assessment (nonresearch). All participating communities were educated about the importance of evaluations and participants were assured that there would be no repercussions for refusing to participate. Then consent was obtained from the parents and guardians of all participants, while assent was obtained from the participating children.

## 3. Results

### 3.1. Mass Treatment

**Figure 3:**
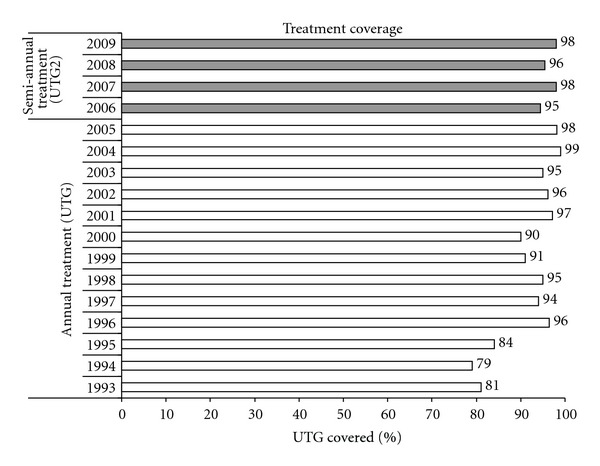
History of percent treatment coverage with ivermectin annually 1993 to 2005 (UTG), and twice yearly (UTG2) from 2006–2010 in Wadelai onchocerciasis focus, Uganda.

Treatment coverage remained high even as the CDTI program expanded geographically and moved to twice-per-year treatment during the period 2006–2010. Figures 2 and 3 show increasing numbers of treatments, and attaining and sustaining the treatment coverage of at least 90% of UTG and UTG2.

### 3.2. Skin Snip (Microscopy) and Nodule Rates

The results showed 100% reduction in baseline microfilariae (mf) skin prevalence. In the baseline survey, mf was 24% in 50 adults, while in the follow up study, mf was 0% in 513 adults assessed. Also, a reduction of 97.8% in clinical onchocercoma (nodule) from the baseline prevalence of 50% in 30 adults to 1.1% in 513 adults (Table 1). A single section of one of the nodules collected revealed a degenerated female filarial worm; the other nodules were not onchocercomas. Thus, the corrected nodule rate was 1 of 513 persons (0.2%), and the presence of viable worms in nodules rate was 0%.

### 3.3. Serological Assessment

 Of the 3011 children tested, three putative positives were obtained. Of these putative positives, only one skin snip collected from a child above 10 years of age was positive in the -150 PCR assay, confirming a 0.03% infection rate (Table 2).

### 3.4. Entomological Assessments

Along the main River Ora, where five sites were surveyed only three *Potamonautes niloticus* crabs caught, were negative for the larval stages of *S. neavei*. Further searches for immature *S. damnosum s.l* in the river were unsuccessful. In February 2010, a brief but intense search for *S. neavei* and *S. damnosum s.l*, made over three days along the main River Ora, identified only three sites, for a total of 6 crabs. None were infested with stages of *Simulium.* This was a very low catch and included no juvenile crabs, suggesting that the crab population was heading for extinction, possibly due to environmental changes. Landing fly catches made at 2 sites for 8 man days yielded no *Simulium* flies. The river near the community of Oruga East was the only place suitable for breeding *Simulium* flies within the focus. No *Simulium* larvae were found and no landing flies were recorded. Throughout the focus, interviews with residents revealed no knowledge of *Simulium *fly biting, although everyone interviewed was conversant with the appearance of tsetse flies. Upstream of the focus, only 11 crabs were caught at 2 sites in 12 hours trapping. None were infested with *S. neavei* larval stages. 

## 4. Discussion

The data supports the conclusion that transmission of onchocerciasis in Wadelai focus has been interrupted and the disease eliminated. The 2009 evaluations showed no active *Onchocerca volvulus* in the nodules and no microfilaria in skin snips from 513 persons, and only 1 of 3011 children (<0.1%) was antibody positive for OV16 antigen. The child who was confirmed positive for a patent infection by PCR was from an older age group that could represent an old exposure. Rapid entomological surveys in 2008 and 2010 for *Simulium *vectors were negative, and people did not recognize black flies as nuisance biters. Based on these (albeit noncomprehensive) entomological findings, the disappearance of onchocerciasis from Wadelai may not be entirely attributed to ivermectin distribution. However, it was likely aided by the disappearance or substantial reduction of the *Simulium* population due to environmental changes.

 Elimination of onchocerciasis in Wadelai was launched at a time before systematic entomological assessment through *Simulium* fly collection and crab infestation through crab trapping for *S. neavei* became a standard in Uganda. Therefore we cannot report baseline data or long-term attempts to collect *S. neavei* and *S. damnosum s.l* vector flies within the focus. This is one of the many challenges that were faced when the decision to change from control to elimination strategy was launched. Entomological assessment of onchocerciasis transmitted *S. neavei *is done by ascertaining breeding through crab trapping and determination of the level of crab infestation with young stages of the fly. *S. neavei *young stages (larvae and pupae) live in a phoretic association on freshwater crabs. Therefore, no infestation with young stages of the fly implies no *S. neavei,* and hence absence of *S. neavei* transmitted onchocerciasis. As mentioned above, lack of knowledge of black flies among the Wadelai residents is an additional and strong evidence that unexplained changes may have resulted in loss of suitable vector habitat and interruption of transmission years earlier.

 Models of published field data from the Americas have demonstrated that twice yearly treatment with ivermectin can eliminate onchocerciasis within 6.5 years [22]. Twice-per-year treatment is the strategy for elimination of transmission in the Americas. The data reported here were collected as a result of a pilot study developed by the Ugandan Ministry of Health (MOH), in order to test the feasibility of implementing an intensive treatment strategy in a small and well-defined focus. The purpose of this study was to determine the operational challenges of implementing twice yearly ivermectin administration and expanding treatment geographically to include all potentially infected communities regardless of baseline endemicity [23]. Experts had questioned practicability of expanded twice-per-year treatment in CDTI projects in Africa [24]. The Wadelai focus project provided evidence that CDTI can be rapidly expanded, and that community ivermectin distributors can distribute ivermectin twice a year with coverage of at least 90% of the UTG population in all treatment cycles.

The Uganda Onchocerciasis Elimination Expert Advisory Committee (UOEEAC) has met since 2008 as an advisory committee to the Ministry of Health, Uganda, and provided recommendations on where and when to halt interventions. At its third meeting in August 2010, UOEEAC considered the Wadelai focus had met the Ugandan national guidelines for interruption of transmission (which are based on the 2001 WHO criteria for certification of elimination of onchocerciasis [15, 16, 25, 26]. The UOEEAC concluded that onchocerciasis had been eliminated from Wadelai, and the ministry of health subsequently accepted that recommendation. However, UOEEAC recommended strengthening of entomological activities during a three-year post-treatment surveillance (PTS) phase through collection, processing and analysis of *S. damnosum s.l* if any, and crab trapping for possible infestation with larval stages of *S. neavei* [23]. The advisory committee also recommended the implementation of the standard protocol for monthly fly collection and crab trapping early during implementation of elimination activities in onchocerciasis foci under the elimination strategy. This would ensure proper tracking of entomological indicators in every onchocerciasis focus under elimination strategy, a missed opportunity in Wadelai.

It was also from Wadelai experience that UOEEAC recommended assessment of children younger than 10 years of age for antibodies against OV16. Although, O-150 polymerase chain reaction (PCR) analysis is highly sensitive and specific, it would not be affordable for routine application in a sample of at least 3000 children needed to calculate a prevalence rate with a one-sided 95% CI <0.1% if zero positives are found [16]. That is why it has been used in the present study only as a confirmatory test for OV16 recombinant antigen putative positive children.

Based on the fact that only 1 of 6 nodules was histologically an onchocercoma reinforced the poor predictive value of nodule as an indicator of onchocerciasis elimination efforts [27]. Subsequently, the UOEEAC removed nodule rates as indicators of morbidity, transmission, and elimination of onchocerciasis. Therefore the experience gained from Wadelai has not only been a precursor for the Uganda countrywide onchocerciasis elimination policy, but has also influenced the national guidelines for certification of onchocerciasis elimination in Uganda [23, 26]. 

CDTI interventions cannot be stopped in Wadelai, where the situation is complicated by coendemicity with lymphatic filariasis (LF). Accordingly ivermectin and albendazole will continue to be administered annually to halt transmission of *Wuchereria bancrofti*, and, despite the status of onchocerciasis described above, no action could be taken to halt ivermectin distribution. As elimination of onchocerciasis becomes more of a prospect in Africa, coordination of onchocerciasis and LF elimination efforts is essential in foci such as Wadelai where co-endemicity exists so that elimination of both diseases can be achieved in an integrated fashion, allowing similar interventions to be halted at the same time [28].

## Figures and Tables

**Table 1:** Prevalence of skin snips (microscopy) and nodules in 2009.

Community	No. examined	% mf	% nodule
		prevalence	prevalence^∗∗^
Aguu West	88	0	2.3
Olimbuni/Aroga^∗^	75	0	0
Aroga Leba	70	0	0
Lwalo	120	0	2.5
Ojigo East	108	0	1.9
Pailo East	52	0	0

Total	513	0	1.12

^∗^Baseline parasitological assessments (Aroga sentinel community) in 1993-mf % positive = 24% of 50 adults and nodule prevalence = 50% of 30 adults.

^∗∗^Only one of six nodules was parasitologically confirmed. The confirmed nodule did not contain any viable adult worms.

**Table 2:** Blood spots from children ≤14 years of age (*n* = 3011) from Wadelai onchocerciasis focus tested for IgG4 antibodies against the OV-16 recombinant antigen in 2009.

Age group	No. screened	No. positive	% positive
1 to 4	1080	0	0
5 to 9	1058	0	0.19
10 to ≤14	873	1	0.11

Total	3011	1	0.03

NB: Only the putative positive individual in age group 10 to ≤14 years was PCR positive by O-150 skin snip PCR, indicating patent infection.
